# Endocrine-Disrupting Chemicals: Some Actions of POPs on Female Reproduction

**DOI:** 10.1155/2013/828532

**Published:** 2013-05-23

**Authors:** Ewa L. Gregoraszczuk, Anna Ptak

**Affiliations:** Department of Physiology and Toxicology of Reproduction, Institute of Zoology, Jagiellonian University, Gronostajowa 9, 30-387 Krakow, Poland

## Abstract

Persistent organic pollutants (POPs), such as polychlorinated dibenzo-p-dioxins (PCDDs) and dibenzofurans (PCDFs), polychlorinated biphenyls (PCBs), and polybrominated ethers (PBDEs), chloronaftalens (PCNs), and bisphenol A (BPA), are stable, lipophilic pollutants that affect fertility and cause serious reproductive problems, including ovotoxic action, lack of ovulation, premature ovarian failure (POF), or polycystic ovarian syndrome (PCOS). Most of the representatives of POPs influence the activation of transcription factors, not only activation of aromatic hydrocarbon receptor (AhR), but also the steroid hormone receptors. This minireview will focus on a variety of PAH activities in oocyte, ovary, placenta, and mammary gland. The complexity and diversity of factors belonging to POPs and disorders of the reproductive function of women indicate that the impact of environmental pollution as an important determinant factor in fertility should not be minimize.

## 1. Introduction

**Figure 1:**
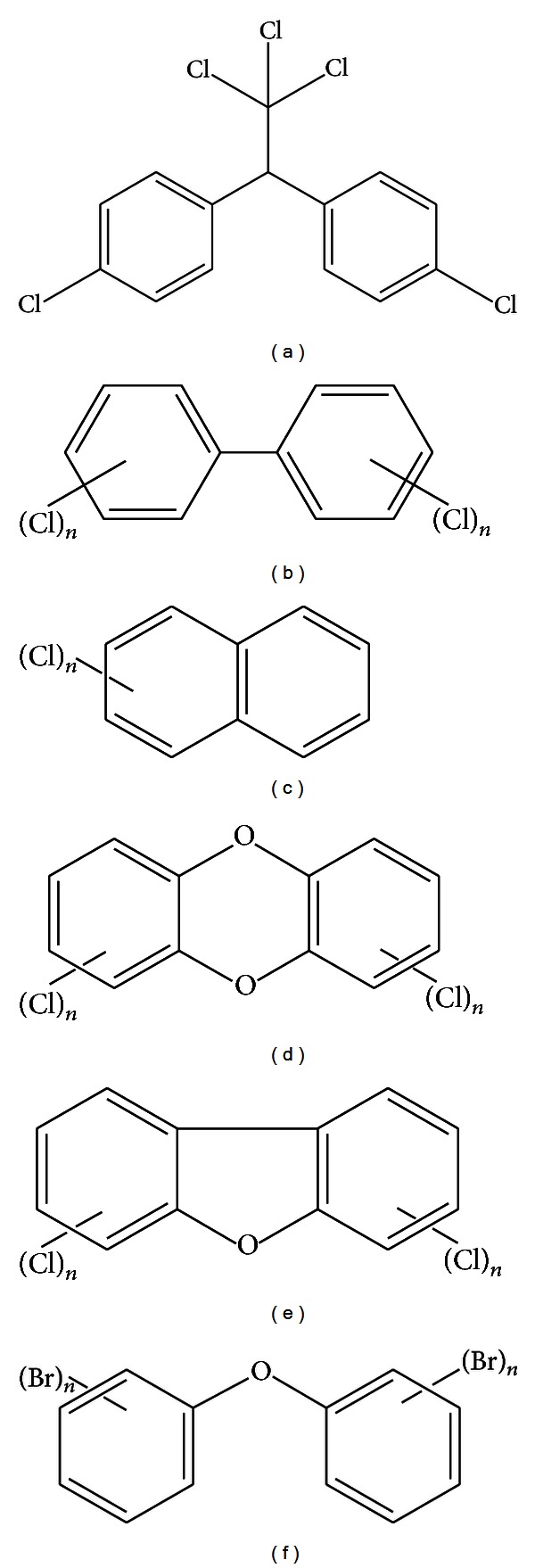
Generic structure of the (a) DDT, (b) PCBs, (c) PCNs, (d) PCDDs, (e) PCDFs, and (f) PBDEs.

 For the past decade, scientists, institutions, governments, and policymakers have warned the general public about serious health hazards associated with chemicals known as endocrine disruptors (EDs). EDs are exogenous compounds that interfere with the synthesis, secretion, transport, metabolism, and/or action of endogenous hormones that are responsible for normal homeostasis, reproduction, and development. Chemicals with hormonal activity can be divided into three main groups: (i) synthetic compounds used in industry and agriculture as well as in consumer products, (ii) synthetic compounds used in pharmaceutical drugs, and (iii) natural compounds present in the food chain (i.e., phytoestrogens, compounds that are structurally similar to estrogen (E2)). Only (i) synthetic compounds used in industry and agriculture and consumer products will be discussed in presented paper. Within this class, compounds can be further subcategorized into those that are persistent in all elements of the environment, bio-accumulative, transportable over long distances, and capable of adversely affecting life forms that reside within short and long distances from the site of contamination. These compounds include dichlorodiphenyltrichloroethane (DDT, a pesticide), polychlorinated biphenyls (PCBs), polychlorinated naphthalenes (PCNs), polychlorinated dibenzodioxins (PCDDs), polychlorinated dibenzofurans (PCDFs), and polybrominated diphenyl ethers (PBDEs) (Figure 1). 

 Because xenobiotics can accumulate in the body for an extremely long period of time (i.e., decades), they can be detrimental to human health even at very low doses. For instance, pesticides and other synthetic compounds used in the 1950s have polluted the air that we breathe, the water that we drink, and the soil that we grow our food in. In addition, many of these compounds are nonbiodegradable. Studies have shown the presence of EDS both in adipose tissue and other organs, in almost all humans and many animals. Human exposure to environmental toxicants is mediated via food and water chains, breathing, and dermal absorption, with approximately 90% of the exposure coming from food. Moreover, several mammalian organs (e.g., ovary, breast, uterus, cervix, bone, muscle, and skin) rely on sex steroids for normal function, and these organs are especially vulnerable to endocrine disruption by environmental toxicants. This chapter will focus only on selected actions or abnormalities. Much of the information on the impact of EDs on human health has come from *in vivo* studies where animals were exposed to a single compound, usually at an acute (i.e., pharmacological) dose; however, humans are exposed to several compounds at unknown concentrations for unknown durations. For example, hospital patients receive an average of six drugs daily (i.e., aspirin, antihistamines, antibiotics, anti-cough syrup, etc.). Food and water may also contain low levels of organic and inorganic compounds (e.g., pesticides and heavy metals) and solvents (e.g., benzene, toluene, and chloroform), and air is filled with hundreds of chemicals (e.g., industrial pollutants, smoke, gasoline vapors, etc.). The ability of these environmental toxicants to affect human health depends on several factors, including interactions between compounds; absorption, metabolism, accumulation, and excretion of compounds; and the ability of some compounds bind to cell receptors which can affect hormone action. Unfortunately, little is known about how these environmental toxicants interact with each other existing in a mixture. It is known that such xenobiotic acts in different ways depending on whether their action is observed on the fetus, newborn babies, or adult individuals, depending on the period during whose body has been exposed to these factors. In many cases, the effect of their actions may be undetected until sexual maturity, especially when exposure occurs during a period of embryonic development or shortly after birth. The female reproductive cycle is a complex process comprised of gametogenesis, embryogenesis, menstruation, ovulation, possible pregnancy, endometrial, and mammary gland changes. Development of the female reproductive system during fetal life determines reproductive success. Women are born with all the oocytes they will ever have through their life, and therefore oocytes are more vulnerable to toxic chemicals than germ cells in men who continue to make more germ cells. 

## 2. Actions on the Oocyte

 Oocyte maturation, the final step of germ cell differentiation, determines reproductive capacity. Follicles are especially susceptible to the adverse effects of environmental toxicants, and developing oocytes may be damaged directly or indirectly by action on follicular cells (i.e., granulosa cells) that may also be vulnerable to endocrine disruption, thereby affecting oocyte function indirectly. A previous study illustrated that human oocytes harvested from follicles with elevated levels of polycyclic aromatic hydrocarbons (PCAHs) had fewer cell divisions after *in vitro* fertilization [1]. Chloroorganic mixtures such as PCBs and DDT, as well as its metabolites, have also been reported to affect puberty, development, and oocyte viability adversely [2]. Some of these studies point to unpredictable changes of translational regulation within the oocyte under the influence of PCBs [3]. Polyspermia (secretion of an excessive amount of semen) has also been demonstrated in cattle exposed to an environmentally relevant mixture of more than 15 organochlorines [4]. Presently, additional research is needed to better understand the molecular mechanisms behind mammalian ovotoxicity caused by exposure to environmental toxicants.

 Ovarian function is controlled by the hypothalamus, pituitary, and autoparacrine factors. Hormone-mimicking compounds can bind to cell receptors, interfere with hormone action, and affect ovarian function. How EDs affect ovarian function is not yet clear, but a disruption in gonadotropin (i.e., follicle stimulating hormone (FSH) and luteinizing hormone (LH)) secretion and feedback mechanisms involving E2 and progesterone (P4) may be involved. Alternatively, EDs may affect ovarian hormone production and oocyte maturation. Damaged oocytes can affect overall hormone production and follicular function, resulting in an endocrinological imbalance (i.e., a decrease in E2 and P4, but an increase in FSH and LH) and ovarian failure. 

## 3. Actions on the Ovary

### 3.1. Disruption of Ovarian Function

 Fertility in sexually mature women depends largely on the maintenance of healthy follicles, and their steady production ensures that an adequate number of follicles reach the antral stage. The stage of development at which the follicles are destroyed determines the influence of these factors on the fertility of women. Complete depletion of the follicle reserve results in irreversible infertility; partial depletion of the follicle reserve results in moderate effects on periodicity. Damage to large follicles can also lead to reversible acyclic disorders that affect hormone production and ovulation. Nevertheless, the effects of POPs on the hypothalamic-pituitary-ovarian (HPO) axis are generally reversible because they do not permanently affect the follicle reserve.

#### 3.1.1. TCDD

TCDD (2, 3, 7, 8-teterachlorodibenzo-para-dioxin) is an isomer of PCDD, and one of the most toxic man-made compounds to pollute our environment. Studies on the mechanism of TCDD action during ovulation indicate a dysfunction in the HPO axis. Misregulation of FSH and LH secretion before ovulation was noted in rats exposed to TCDD. Moreover, gonadotropin-releasing hormone (GnRH) inhibited the surge in FSH and LH secretion, thereby restoring ovulation [5, 6]. TCDD also inhibited FSH secretion and downregulated FSH receptor gene expression [7]. TCDD affected the levels of other hormones as well. For instance, Gore [8] showed methoxychlor to affect GnRH expression in hypothalamic GT1-7 cells. In a study from our laboratory, TCDD inhibited E2 secretion by follicular cells and P4 secretion by luteal cells dose-dependently. These adverse effects on hormone production were mediated in part by enzymes involved in steroidogenic biosynthesis. In luteal cells, TCDD action was independent of E2 receptor stimulation, instead involving the aryl hydrocarbon receptor (AhR) [9]. On a final note, other environmental toxicants (i.e., PCDFs, biphenyls, DDT, and methoxychlor) can also block the surge in LH and FSH secretion during the female reproductive cycle [10, 11].

#### 3.1.2. PCBs

 Polychlorinated biphenyls (PCBs) are the man-made chemicals that may disrupt follicular steroidogenesis either by mimicking natural hormones as agonist or antagonist, altering the pattern of hormone synthesis, modulating hormone receptor affinities or numbers, or by altering enzymes involved in hormone secretion. In our previous study, we showed that the orthosubstituted PCB 153 congener accumulated preferentially in the follicular wall when compared to the nonorthosubstituted PCB 126 congener. 71%, 71.4%, and 30.4% of the total exposure for PCB 153 were in small, medium, and large follicles, respectively (Figure 1). Interestingly, about 70% of PCB153 accumulated in early antral and antral follicles and only 30% in preovultory follicles. The consequence was a reduction in estradiol secretion by early antral and antral follicles and lack of influence on estradiol secretion by preovulatory follicles. Moreover, Moreover, it has been showed that action on estradiol secretion was correlated with action on aromatase activity [12–14]. A similar dose-responsive relationship was reported after exposure of follicular cells to a mixture of organic pollutants [15] or a mixture of PBDEs [16]. 

#### 3.1.3. PBDEs

 Polybrominated dibenzoethers (PBDEs) are persistent and ubiquitous environmental toxicants found at increasing levels in humans and animals. Despite recent bans by the European Union [17], United States, and China on the production of penta- and octa-BDE as well as on the diminished use of PBDE in Japan [18], 2,2′,4,4′-tetra-BDE (BDE-47), 2,2′,4,4′,5-penta-BDE (BDE-99), and 2,2′,4,4′,6-penta-BDE (BDE-100) are the major PBDE congeners present in humans and animals [19]. The literary data related to the effects of PBDE mixture are limited predominantly to commercial mixture DE-71 which contains mainly penta-BDEs (BDE-99 and BDE-100) and tetra-BDE (BDE-47). Zhou et al. [20] and Stoker et al. [21] indicated that DE-71 (a mixture of BDE-99, BDE-100, and tetra-BDE) affected the production of thyroid and sex steroids and the development of reproductive organs [20, 21]. Results from our laboratory have shown an increase in the P4/testosterone (T) ratio but a decrease in the T/E2 ratio, in ovarian follicles suggesting premature luteinization of antral follicles. Removal of the PBDE mixture from cell cultures did not reverse adverse effects [22]. In a follow-up study, we reported changes in the levels of steroidogenic enzymes (e.g., 17*β*-hydroxysteroid dehydrogenase (17*β*-HSD), cytochrome P450, family 17, subfamily A, polypeptide 1 (CYP17), and aromatase (CYP19)) by PBDE congeners 47, 99, and 100 [23]. Last published data showed fast activation of CYP2B1/2, and late activation of COMT (with a very low basal SULT1A activity) in ovarian follicles by BDE-47 indicates a possible action of locally produced hydroxylated metabolites prior to their detoxification [24]. Additionally, it have been showed that 5-OH-BDE-47 and 6-OH-BDE-47 have a different mechanism of action in ovarian follicles from their parent compound and lead to an increase in estradiol secretion. The metabolites stimulate aromatase expression and activity, while the parent compound increases androgen production and stimulation of 17*β*-HSD protein expression and activity [25].

#### 3.1.4. PCNs

 Polychlorinated naphthalenes (PCNs) are members of a large and diverse group of compounds with several industrial applications. PCNs occur as mixtures of congeners sold under various trade names (e.g., Halowax, Nibren Wax, and Seekay Wax) [26]. The toxicological characteristics of PCNs are similar to those caused by PCBs, PCDDs, and PCDFs [27, 28]. Presently, there are little data on the toxicity of PCNs in experimental animals, and our recently published data was the first showed direct action on ovarian function. We showed an increase in basal testosterone secretion in all doses used, with the highest stimulatory action of the smallest dose, which was accompanied by a parallel decrease in basal estradiol secretion, induced by Halowax 1051 suggesting androgenic properties of Halowax 1051. As a mechanism we propose direct stimulatory action on 17*β*-HSD activity and protein expression, enzyme responsible for testosterone synthesis and inhibitory action on CYP19 activity, and enzyme responsible for conversion testosterone to estradiol [29]. It should be taken into consideration that as in the case of BDE-47 [24], the effects of exposure to PCNs may be due to the side effects of PCN metabolites. Examining the effects of the Halowax 1051 on phase I (CYP1A1) and phase II (SULT1A and COMT) enzyme activities and expression in cultured ovarian follicles we showed fast activation of enzymes involved in phase I and concurrent inhibition of enzymes involved in phase II metabolism confirming our suggestion that the observed effects of Halowax 1051 are partially result from the action of metabolites formed locally in ovarian follicles [30].

#### 3.1.5. HCBz

 Data on the effects of hexachlorobenzene (HCBz) on reproduction are scant. While HCBz is toxic to humans [31, 32] and animals [33], information on the effects of HCBz in ovarian steroidogenesis is limited despite data that showed an increase in the P4 serum level in superovulated rats exposed to HCBz [34]. Treatment of Cynomolgus monkeys with HCBz, on the other hand, decreased the P4 serum level and unaltered the E2 serum level during the luteal phase. In a recently published study, we determined *in vitro* accumulation of hexachlorobenzene (HCBz) and pentachlorobenzene (PeCBz) in porcine ovarian follicles, the effect on steroidogenesis, and the expression of enzymes responsible for steroid synthesis [35]. We showed that sixty percent of the HCBz and almost 100% of the PeCBz that was added to the culture medium accumulated in ovarian tissue, and only 1% of each was found in the medium. Moreover, we showed inhibitory HCBz effect and stimulatory PeCBz effect on testosterone and estradiol secretion. As a conclusion we mentioned that the greater exposure to an estrogenic action of PeCBz than antiestrogenic HCBz would be a consequence of the preferential accumulation of PeCBz in the ovarian follicles. Another situation was observed in human placental tissue. Both HCBz and PeCBz did not accumulate in placental tissue. We showed that HCBz by fast activation of I and II phase metabolism is probably metabolized to PeCBz in placental tissue (Gregoraszczuk et al., unpublished data).

#### 3.1.6. A Mixture of Environmental Toxicants

 Toxicants usually occur in a mixture of several toxicants, making it difficult to predict adverse effects on human health. Younglai et al. [36] demonstrated human follicular fluid obtained from women to contain 1, 1-dichloro-2, 2-bis (p-chlorophenyl)-ethylene (p, p′-DDE), mirex (a pesticide), hexachloro-ethane 1, 2, 4-trichlorobenzene, and numerous PCBs (i.e., PCBs 49, 153, and 180). Of these, p, p′-DDE was the most frequently detected ED in follicular fluid and serum. Several environmental toxicants are ER agonists, and they include estrogenic steroids (natural and synthetic), phyto- and mycoestrogens, and xenoestrogens (e.g., pesticides, plasticizers, and alkylphenols). In addition to simple additive effects [37], interactions between different chemicals in a mixture may result in either a weaker (antagonistic) [38] or stronger (synergistic, potentiated) combined effect than would be expected from knowledge about the toxicity and mode of action of each individual compound. These interactions may occur during toxicant uptake, distribution, metabolism, and/or excretion (i.e., toxicokinetic phase), or during toxicant binding to receptors and cellular targets (i.e., toxicodynamic phase) [39, 40]. A mixture of xenoestrogens may alter the way a cell responds to endogenous estrogens. For instance, xenoantiestrogens found within mixtures can inhibit endogenous E2. Moreover, some estrogenic compounds exert their effects, not by binding to ER but rather by binding to estrogen plasma transport proteins, resulting in an increase in free endogenous E2. Nevertheless, mixtures containing both xenoestrogens and endoantiestrogens may have no adverse effects [41]. In our previously published data, to determine which compounds within a PCB-DDT-DDE mixture stimulated E2 secretion, we exposed cells to PCBs 118, 138, 153, and 180, DDT, and DDE alone or in different combinations. Interestingly, DDT and DDE affected E2 secretion [42], and these results are in agreement with another series of experiments that used mixtures of PBDEs [43]. In the next experiments, using Western blot analysis indicated that PCBs mixture (“Marine mix”) is an inducer of AhR and mixed-type CYP inducer (CYP1A1 and CYP2B1) while PBDEs mixture (“Mjosa mix”) is an inducer of ER*β* and CYP2B [44]. 

### 3.2. Pathology of the Ovary

#### 3.2.1. Early Puberty

 Early puberty is a recent growing concern as there are reports of many girls reaching their first menstruation and developing breasts earlier in life than was the case 40 years ago [45]. Early puberty associates with polycystic ovarian syndrome (PCOS), obesity, breast cancer, depression, and a number of social challenges such as experimentation with sex, alcohol, or drugs at a younger age. Moreover, earlier menarche and thelarche ages have been reported in girls after exposure to PCBs, PBBs, DDT, and/or phthalate esters [46, 47], and precocious puberty has been observed following exposure to DDT metabolites [48].

#### 3.2.2. Polycystic Ovarian Syndrome (PCOS)

The acyclicity of the syndrome is linked with the hyperfunctioning of theca and hypofunctioning of granulosa cells. PCOS also associates with other processes (e.g., neuroendocrine function and ovarian steroidogenesis) and diseases (e.g., insulin resistance, and obesity) that are regulated by hormonal and metabolic factors. Thus, exposure to environmental toxicants may indeed contribute to the pathogenesis of PCOS. For instance, women with PCOS have higher levels of bisphenol A (BPA, a plastics additive) [49] and testosterone which is consistent with the decreased clearance of BPA that is often observed [50]. Although a cause-and-effect relationship has not been established, the role of environmental toxicants in the pathogenesis of PCOS is worthy of further consideration.

#### 3.2.3. Premature Ovarian Failure (POF)

POF, the cessation of normal ovarian function before the age of 40, occurs in approximately 1% of women of reproductive age [51]. The underlying causes of POF are largely known in most cases, and any factor that can decrease the ovarian reserve can result in POF. For instance, disruption of germ cell migration from the genital ridge to the developing gonad results in ovarian dysgenesis and POF. In addition, adult and *in utero *exposure of mice to BPA resulted in oocyte damage [52, 53], whereas exposure of women to cigarette smoke decreased fertility, *in vitro *fertilization (IVF) success rates, and the ovarian reserve resulted in earlier menopause and increased miscarriage rate [54]. In another study, exposure of rats to TCDD *in utero *and throughout reproductive life resulted in premature reproductive senescence [55]. Endocrine disruption caused by acute exposure to environmental toxicants such as the AhR agonist TCDD suggests that AhR-mediated apoptosis of oocytes may be involved. 

## 4. Hormone-Dependent Cancer

### 4.1. Breast Cancer

 Breast cancer is the most frequent neoplasm affecting women residing in Western countries and is the second leading cause of death [56]. The general population is exposed to several hormonally active compounds on a daily basis. The majority of these compounds are xenoestrogens (e.g., PCAHs, pesticides, PCBs, PBDEs, DDT, selected drugs, fungicides, phytoestrogens, mycotoxins, BPA, and phthalates), and they can possess estrogenic action, affect estrogen levels, and/or bind to ERs [57].

#### 4.1.1. Recent Findings on PCBs

 The role of PCBs in breast cancer has been investigated intensively. Data suggest that a correlation may exist between high levels of PCBs in mammary tissues or sera and breast cancer risk [58], while another study reported no association [59]. High PCB levels upregulated CYP expression [60], suggesting that PCB metabolites may be critical for the pathogenesis of breast cancer. Likewise, Pang and colleagues [61] reported that exposure of MCF-7 human breast cancer cells to PCBs 81, 126, and 39 increased CYP1A1 and CYP1B1 mRNA levels, resulting in the formation of E2 metabolites. We demonstrated PCB 3 to induce and to be a substrate for CYP1A1in MCF-7 cells [62]. On the other hand, others have shown PCBs 52 and 77 to induce oxidative damage (i.e., DNA strand breaks) in ER*α* (−)/MDA breast cancer cells but not in ER*α* (+)/MCF-7 cells [63], suggesting that the ER*α* receptor may play a protective role in breast cancer. Moreover, PCBs can interfere with the balance between proliferation and apoptosis. Radice et al. [64] demonstrated those PCB congeners such as PCBs 101, 118, 138, 153, and 180 increased proliferations of MCF-7 cells. Our published data demonstrated that from investigated congeners (118, 138, 153, and 180), PCB138 and 153 had the highest stimulatory effects on basal MCF-7 cell proliferation as well as the highest inhibitory actions on basal caspase-9 activity. Moreover, we showed that PCBs 138 and 153 contribute to the action of endogenous 17*β*-estradiol on cell proliferation and apoptosis in the breast cancer cell line MCF-7 [65]. *In vivo* experiments have also shown PCBs to increase metastasis by triggering the production of reactive oxygen species (ROS), thereby activating the Rho-associated protein kinase (ROCK) signaling pathway [66] or vascular endothelial growth factor (VEGF) overexpression that stimulates endothelial hyperpermeability and transendothelial migration of cancer cells [67].

#### 4.1.2. Recent Findings on PBDEs

 Data on the effects of PBDEs on breast cancer are scant. PBDEs trigger micronucleus formation and proliferation in MCF-7 cells [68]. An increase in MCF-7 cell proliferation by DE-71 (a mixture of BDE-47, -99, -100, -153, and -154) was also reported by Mercado-Feliciano and Bigsby [69]. PBDE-209 also induced MCF-7 cell proliferation by affecting critical steps of the cell cycle [70]. At the molecular level, PBDE-209 triggered protein kinase C *α* (PKC*α*) and ERK1/2 phosphorylation. On the other hand, Kwiecińska et al. [71] have shown no changes in MCF-7 cell proliferation following exposure to BDE-47, -99, -100, and -209; however, apoptosis was inhibited by decreasing caspase-9 activity in these cells [71]. Resistance to apoptosis associates with tumorigenesis as it enables tumorigenic cells to expand even in a stressful environment. Thus, additional studies are needed to determine if exposure to PBDEs can indeed cause breast cancer.

#### 4.1.3. Recent Findings on Bisphenol A (BPA)

 Laboratory studies in rodents suggest a link between BPA exposure and breast cancer incidence. When rodents were exposed to BPA early in life, there were changes in mammary gland morphogenesis and tumor susceptibility [72–74]. These findings are supported by *in vitro* data which demonstrated BPA to induce transformation of MCF-10F human breast cancer cells. These cells formed tubule-like structures when cultured in 3D collagen matrix, but spherical masses were noted after BPA treatment [75], leading the authors to conclude that BPA produces adducts or ROS which can inadvertently introduce a variety of DNA modifications and cause breast cancer. Others have shown BPA to stimulate proliferation but to inhibit apoptosis in MCF-7 cells [76–78]. BPA may also induce breast cancer cell proliferation by upregulating cell cycle genes and downregulating antiproliferative genes, especially genes that control the G1/S transition via ER*α* signaling [79]. 

### 4.2. Ovarian Cancer

Ovarian cancer is the most prevalent type of gynecological cancer affecting women residing in Western countries. As more than 60% of tumors are diagnosed at stage III and certain forms of cancer are very aggressive, ovarian cancers are associated with a high mortality. While most cells undergo neoplastic transformation, including germ cells, granulose, and stromal cells, approximately 90% of tumors are derived from the ovarian surface epithelium (OSE). Similar to breast cancer, hormonal factors such as estrogen and xenoestrogens have been linked to ovarian cancer [80, 81]; however, the role of environmental toxicants in ovarian cancer requires further study.

#### 4.2.1. Recent Findings on PCBs and PBDEs

 The role of PCBs in the initiation and progression of ovarian cancer is unknown. Studies in adult C57BL mice showed that orally administered PBDEs-47, -85, and -99 to accumulate in the liver, adrenal cortex, and ovary in adult C57BL mice [82] suggest a possible carcinogenic activity, especially in light of research showing that exposure of CHO and OVCAR-3 cells to PBDE-209 initiated S and G2/M phases of the cell cycle, respectively [83].

#### 4.2.2. Recent Findings on BPA

Several *in vitro *studies have shown BPA to induce chromosomal aberrations in CHO cells [84, 85], a common genetic alteration in cancer. Moreover, ovarian cyst formation was observed in mice treated neonatally with BPA [86]. Ovarian cancer may stem from incessant ovulation, which may be linked to the formation of cysts that are frequently found in perimenopausal women. In a study from our laboratory, we demonstrated an increase in proliferation in OVCAR- 3 cells treated with BPA. Specifically, we showed BPA to promote the cell cycle by upregulating the expression of cyclin D1, CDK4, E2F1, E2F3, and PCNA (a mediator of G1 to S-phase progression) and cyclin A (a mediator of G2-phase progression to mitosis), but by downregulating the expression of p21WAF1/CIP1, Weel-1, and GADD45*α* [87]. Additionally, we demonstrated a decrease in the expression of proapoptotic genes (i.e., FAS, FADD, RAIDD, caspase-8, -10, -3, -6, and 7, CAD, Bax, Bak, Bok, and Apaf-1) but an increase in the expression of prosurvival genes (i.e., Bcl-x and Mcl-1). BPA also activates a caspases-independent apoptotic pathway by inducing endonuclease G gene expression. Also, Hwang et al. [88] using microarray analysis increased mRNA levels of E2-responsive genes in ER-positive ovarian cancer BG-1 cells under the influence of BPA. In a subsequent study, we showed BPA to trigger phosphorylation of Stat3, ERK1/2, and Akt in OVCAR-3 cells [89]. Cited results of research indicated that BPA acting as a mitogen as well as an antiapoptotic factor may be an additional factor responsible for ovarian cancer.

## 5. Conclusions

In recent years there has been a growing evidence that exposure to chemicals in the environment poses a serious threat to human and animals reproduction via disrupting effects on endocrine function. Despite the fact that these substances are persistent, they may be metabolized into more toxic compounds than the parent molecule in endocrine organs. This endocrine disrupting chemicals (EDCs) adversely affect health and reproduction even at very low concentrations and may exert their effects on the embryo and fetus. The complexity and diversity of factors belonging to EDCs, its direct action on the ovary, and disorders of the reproductive function of women indicate that the impact of environmental pollution as an important determinant factor in fertility should not be minimize. Current estimates of cancer risk in humans do not account properly for transplacental and environmental (including occupational) exposure to xenoestrogens. It is important to reevaluate the role of xenoestrogens in cancer development using new approaches that better reflect the complexity of carcinogenesis. Testing new compounds before they are allowed to use should be expanded to determine their effect on the endocrine system, in order to assess the hormonal activity. In addition, attention should be directed towards dose-response relationships in environmental toxicology. Such studies can provide useful information that might have a significant impact on the strategies for risk assessment of toxic substances.
